# The **COPE**-Trial—**C**ommunicating prognosis to parents in the neonatal ICU: **O**ptimistic vs. **PE**ssimistic: study protocol for a randomized controlled crossover trial using two different scripted video vignettes to explore communication preferences of parents of preterm infants

**DOI:** 10.1186/s13063-021-05796-3

**Published:** 2021-12-06

**Authors:** Fiona A. Forth, Florian Hammerle, Jochem König, Michael S. Urschitz, Philipp Neuweiler, Eva Mildenberger, André Kidszun

**Affiliations:** 1 Division of Neonatology, Center for Pediatric and Adolescent Medicine, University Medical Center of the Johannes Gutenberg-University Mainz, Langenbeckstrasse 1, 55131 Mainz, Germany; 2 DFG-Research Training Group “Life Sciences – Life Writing”, Institute for the History, Philosophy and Ethics of Medicine, University Medical Center of the Johannes Gutenberg-University Mainz, Am Pulverturm 13, 55131 Mainz, Germany; 3 Department of Pediatric and Adolescent Psychiatry and Psychotherapy, University Medical Center of the Johannes Gutenberg-University Mainz, Langenbeckstrasse 1, 55131 Mainz, Germany; 4 Division of Pediatric Epidemiology, Institute of Medical Biostatistics, Epidemiology and Informatics (IMBEI), University Medical Center of the Johannes Gutenberg-University Mainz, Obere Zahlbacher Strasse 69, 55131 Mainz, Germany; 5 Journalistisches Seminar, Johannes Gutenberg-University Mainz, Alte Universitätsstrasse 17, 55116 Mainz, Germany; 6 Division of Neonatology, Department of Pediatrics, Inselspital, Bern University Hospital, University of Bern, Freiburgstraße, CH-3010 Bern, Switzerland

**Keywords:** Neonatal ICU, Physician-parent communication, Parent-centered communication, Parent-centered research, Prognosis, Uncertainty, Message framing, Optimism, Pessimism

## Abstract

**Background:**

One of the numerous challenges preterm birth poses for parents and physicians is prognostic disclosure. Prognoses are based on scientific evidence and medical experience. They are subject to individual assessment and will generally remain uncertain with regard to the individual. This can result in differences in prognostic framing and thus affect the recipients’ perception. In neonatology, data on the effects of prognostic framing are scarce. In particular, it is unclear whether parents prefer a more optimistic or a more pessimistic prognostic framing.

**Objective:**

To explore parents’ preferences concerning prognostic framing and its effects on parent-reported outcomes and experiences. To identify predictors (demographic, psychological) of parents’ communication preferences.

**Design, setting, participants:**

Unblinded, randomized controlled crossover trial (RCT) at the Division of Neonatology of the University Medical Center Mainz, Germany, including German-speaking parents or guardians of infants born preterm between 2010 and 2019 with a birth weight < 1500 g. Inclusion of up to 204 families is planned, with possible revision according to a blinded sample size reassessment.

**Intervention:**

Embedded in an online survey and in pre-specified order, participants will watch two video vignettes depicting a more optimistic vs. a more pessimistic framing in prognostic disclosure to parents of a preterm infant. Apart from prognostic framing, all other aspects of physician-parent communication are standardized in both videos.

**Main outcomes and measures:**

At baseline and after each video, participants complete a two-part online questionnaire (baseline and post-intervention). Primary outcome is the preference for either a more optimistic or a more pessimistic prognostic framing. Secondary outcomes include changes in state-anxiety (STAI-SKD), satisfaction with prognostic framing, evaluation of prognosis, future optimism and hope, preparedness for shared decision-making (each assessed using customized questions), and general impression (customized question), professionalism (adapted from GMC Patient Questionnaire) and compassion (Physician Compassion Questionnaire) of the consulting physician.

**Discussion:**

This RCT will explore parents’ preferences concerning prognostic framing and its effects on physician-parent communication. Results may contribute to a better understanding of parental needs in prognostic disclosure and will be instrumental for a broad audience of clinicians, scientists, and ethicists.

**Trial registration:**

German Clinical Trials Register DRKS00024466. Registered on April 16, 2021.

**Supplementary Information:**

The online version contains supplementary material available at 10.1186/s13063-021-05796-3.

## Administrative information

Note: This document is based on the SPIRIT guidelines [1]. The numbers in curly brackets in this protocol refer to SPIRIT checklist item numbers. The order of the items has been modified to group similar items (see https://www.equator-network.org/reporting-guidelines/spirit-2013-statement-defining-standard-protocol-items-for-clinical-trials/). A completed SPIRIT checklist is included as additional file [see Additional file 1].

**Table:** 

Title {1}	The **COPE**-Trial – **C**ommunicating prognosis to parents in the neonatal ICU: **O**ptimistic vs. **PE**ssimistic: A randomized controlled crossover trial using two different scripted video vignettes to explore communication preferences of parents of preterm infants
Trial registration {2a and 2b}.	2a The trial is registered at the German Clinical Trials Register “Deutsches Register Klinischer Studien (DRKS)” (www.drks.de; trial registration number: DRKS00024466). 2b The register (DRKS) collects all items from the World Health Organization Trial Registration Data Set.
Protocol version {3}	Version 3.0, February 05^th^, 2021
Funding {4}	The research project is funded in part by the DFG-Research Training Group 2015/2 „Life Sciences **–** Life Writing”, University Medical Center of the Johannes Gutenberg-University Mainz, Mainz, Germany. Types of funding: The funding consists of a person-related funding within the framework of a PhD-fellowship (fellow: cand. med. F. A. Forth). Additional project-related costs (information material for study participants including shipping material and charges, processing costs for the ethics proposal) are covered within the scope of the fellowship.
Author details {5a}	[1]Division of Neonatology, Center for Pediatric and Adolescent Medicine, University Medical Center of the Johannes Gutenberg-University Mainz, Mainz, Germany [2]DFG-Research Training Group “Life Sciences – Life Writing”, Institute for the History, Philosophy and Ethics of Medicine, University Medical Center of the Johannes Gutenberg-University Mainz, Mainz, Germany [3]Department of Pediatric and Adolescent Psychiatry and Psychotherapy, University Medical Center of the Johannes Gutenberg-University Mainz, Mainz, Germany [4]Division of Pediatric Epidemiology, Institute of Medical Biostatistics, Epidemiology and Informatics (IMBEI), University Medical Center of the Johannes Gutenberg-University Mainz, Mainz, Germany [5]Journalistisches Seminar, Johannes Gutenberg-University Mainz, Mainz, Germany [6]Division of Neonatology, Department of Pediatrics, Inselspital, Bern University Hospital, University of Bern, Bern, Switzerland §Corresponding author: Fiona Antonia Forth E-Mail: fionaforth@uni-mainz.de Department Phone: + 49 6131 / 17-9538
Name and contact information for the trial sponsor {5b}	DFG-Research Training Group 2015/2 “Life Sciences – Life Writing”, Institute for the History, Philosophy and Ethics of Medicine, University Medical Center of the Johannes Gutenberg University Mainz Street: Am Pulverturm 13 Postal zip code: 55131 City: Mainz Country: Germany
Role of sponsor {5c}	The funder is not involved nor has any responsibility in the study design; the collection, management, analysis, and interpretation of data; writing of the report; decision to submit the report for publication. They will not have ultimate authority over any of these activities.

## Introduction

### Background and rationale {6a}

The birth of a very low birth weight infant (VLBWI) is associated with numerous challenges, even for experienced neonatologists. This can be due to the medical uncertainties, ethical controversy, and need for timely interventions related thereto [2, 3]. In addition, the setting of a neonatal intensive care unit (NICU) places high demands on parents [3, 4]. Having recently assumed their new role, they are expected to make vital decisions on behalf of their newborn (so-called surrogate decision-making), decisions that equally affect their own future as well as that of their child. This is one of the reasons why some parents describe the experience of prematurity as one that can even be perceived as a life transformation [3].

One challenge prematurity entails for physicians and parents alike is the disclosure of prognoses, i.e., communicating uncertainty. As prognostic disclosure enables the stakeholders to make realistic therapeutic decisions that are appropriate to their situation (here: a premature infant’s situation), it should be an integral part of communication between physicians and patients (here: parents) [5]. Prognoses, however, are based on scientific knowledge (medical evidence) and medical experience. They represent a statistical or frequency-based statement about the probability of an illness trajectory, are subject to individual assessment, and hence, will always remain uncertain with regard to the individual. It can be assumed that it is a major challenge for parents to cope with the uncertainty concerning the future of their preterm infant and their family. For physicians, on the other hand, prognoses entail the difficult task to deduce a personal probability concerning the respective premature infant’s future prospect from a frequent probability [6]. Moreover, it is the physicians’ responsibility to communicate the respective prognosis and the associated uncertainty to parents. In doing so, physicians are expected to be realistic, i.e., neither too optimistic nor too pessimistic.

In addition, previous studies have concluded that prognostic disclosure should strike a balance between providing a concrete prognosis through honesty and communicating hope in a realistic way. Researchers postulate that this can be achieved by imparting medical empathy as well as by reassuring parents that they will not be abandoned and that the attending physician is available for queries [7, 8]. Consequently, successful physician-parent communication not only requires medical expertise, but also the physicians’ interpersonal competence to perceive the needs and preferences of the parents.

Studies in oncology have shown that physicians initiate and proportionally dominate communication regarding prognosis [9, 10]. Prognoses are mostly communicated vaguely and with a focus on medical facts that are difficult for laypersons to understand [9–12]. This conflicts with the desire of parents of critically ill children for a clear communication of a specific prognosis as well as for explicit assertions concerning the impact of the prognosis on their child’s quality of life. This way of communication mainly succeeds when communicating good news, whereas conveying unfavorable prognoses is much more challenging [13].

Moreover, studies indicate that medical professionals tend to be rather (overly) optimistic than pessimistic when framing unfavorable prognoses [11, 12, 14]. Optimistic framing of bad news is often understood (by physicians) as an attempt not to generate a sense of hopelessness in patients (here: parents) [15, 16]. Hope, however, is complex and multimodal [17]. Studies indicate that despite an unfavorable prognosis, it is not unrealistic for parents to hope for a certain outcome to be achieved in their own child, even if they do not expect, i.e., cannot be optimistic, that it will occur [18]. Parents can thus hope that in the individual case of their child the personal probability of the occurrence of the prognosis deviates from the frequent probability in the positive sense for the health and quality of life of the child, even if they are not optimistic in this respect. Accordingly, it is possible for the consulting physician to preserve parental hope regardless of a prognosis’ severity, however, dependent of the manner of prognostic disclosure [18]. It is known that the realistic delineation of an unfavorable prognosis in its overall severity does not contradict the communication of hope [16, 19, 20]. In fact, honesty promotes a sense of hope as it removes uncertainty and allows parents to make a decision consistent with the child’s situation [21]. Data from other medical disciplines suggest that optimistic advice is initially preferred and experienced as positive, and it also increases the perceived empathy of, confidence in, and trustworthiness of medical staff [22]. Notwithstanding, (overly) optimistic framing can be unbeneficial in other regards. It may hamper a realistic assessment of the actual prognosis by parents and thus may impede making an adequate therapeutic decision [14]. Less optimistic framing, on the other hand, entails greater concordance between medical professionals’ and parents’ assessment of the severity of a prognosis and may form the basis of more realistic therapeutic decisions [14]. Furthermore, particularly when a predicted outcome occurs, less optimistic consultation appears to be more beneficial with regard to the physician-parent relationship as well as concerning parents’ coping with the condition [16, 22]. On the contrary, less optimistic disclosure of information can also be experienced as negative, causing distress and thus complicating communication between the stakeholders [16].

Reviewing the partially contradictory scientific findings from other disciplines, the question arises as to which manner of communication, i.e.,framing, forms the basis of successful communication in the context of a NICU. In this regard, it is also of interest to take a closer look at the parental perspective. It is known that parents want to be asked how to receive information concerning their child, and that the choice of words is important [23]. In most cases, when parents express dissatisfaction with their child’s NICU stay, their child is well cared for, but their own parental needs, often in relation to physician-parent communication, are not adequately addressed [24]. In neonatology, data concerning the framing of prognostic disclosure are scarce [9, 11]. Thus, it is of scientific interest to explore parental preferences regarding prognostic framing and its effects.

### Objectives and primary hypothesis {7}

- Primary objective is to determine whether a more optimistic or a more pessimistic prognostic framing is preferred by parents. The corresponding primary hypothesis is that more parents prefer a more pessimistic prognostic framing to a more optimistic prognostic framing.
- Secondary objectives are to explore the effects of prognostic framing (more optimistic vs. more pessimistic) on parent-reported outcomes and experiences, and to identify demographic and psychological predictors of parental communication preferences. All secondary hypotheses are listed along with the respective outcomes in the “Outcomes {12}” section. In addition, all secondary hypotheses are registered (in German and English) in the German Clinical Trials Register (www.drks.de/DRKS00024466) and can thus be viewed transparently.

### Trial design {8} and study setting (participating centers) {9}

**Fig. 1:**
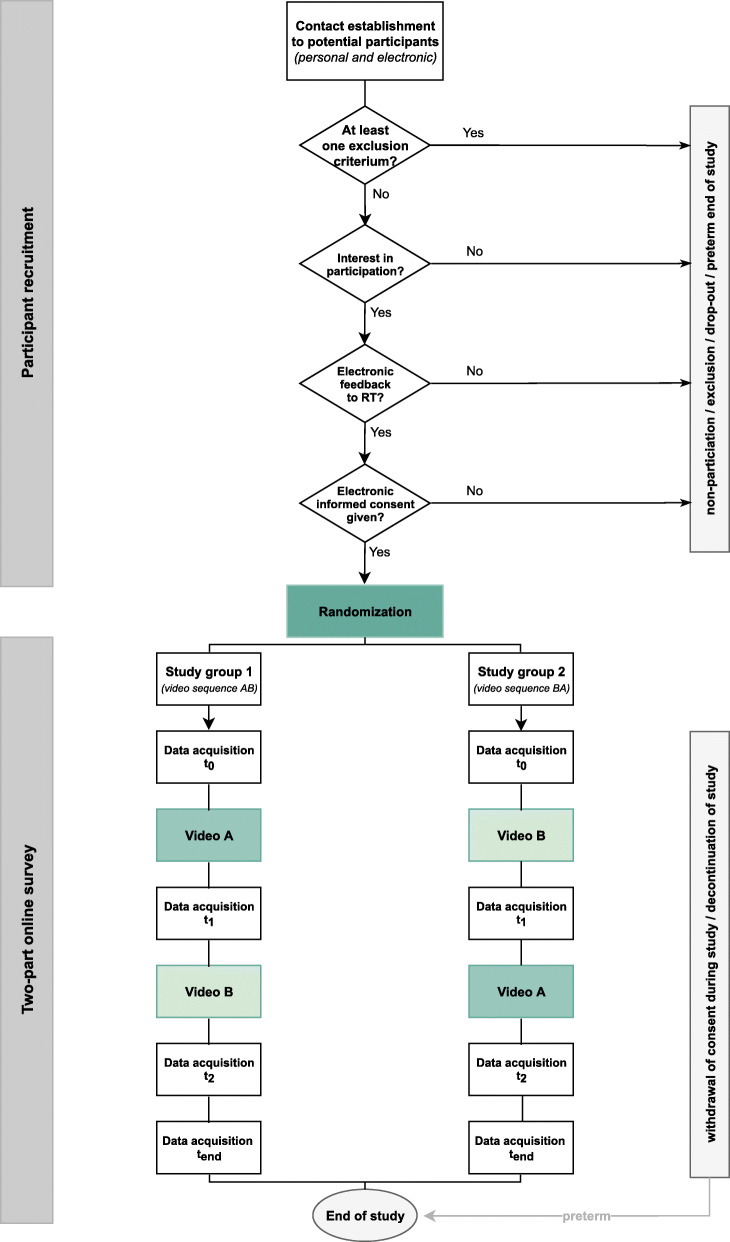
Study sequence and participant timeline, respectively

The COPE-Trial is an unblinded, randomized controlled crossover trial (RCT). The parents’ survey is a single-center study of the Division of Neonatology, Center for Pediatric and Adolescent Medicine, University Medical Center of the Johannes Gutenberg-University Mainz (UMC Mainz). The COPE-Trial is conducted as an online survey with two study groups. Concealed, stratified randomization of participants to the study groups will be performed in blocks of variable length. The online survey consists of a two-part questionnaire (baseline and post-intervention). Embedded in the post-intervention questionnaire and in pre-specified order depending on randomized group allocation, participants will watch two video vignettes (video A and B) depicting a more optimistic vs. a more pessimistic framing in prognostic disclosure to parents of a preterm infant. At baseline (data acquisition *t*_0_) and after each video (outcome measurement including data acquisition *t*_1_, *t*_2_, *t*_end_) participants of both groups complete an identical set of questions. Figure 1 in the “participant timeline {13}” section provides an overview of the study sequence and illustrates the dependence of the sequence of the videos (AB vs. BA) on randomization. Primary outcome is the participants’ preference for either a more optimistic or a more pessimistic framing assessed by the parents’ preference for one of the videos (A or B).

## Methods: participants, interventions, and outcomes

This document is based on the SPIRIT guidelines [1]. The numbers in curly brackets in this protocol refer to SPIRIT checklist item numbers. A completed SPIRIT checklist is included as additional file [see Additional file 1].

## Participants

### Eligibility criteria {10}

#### Inclusion criteria

- Non-bereaved parents or guardians of infants born preterm after December 31st, 2009, but before January 1, 2020, with a VLBW < 1500 g, and postnatal treatment at the UMC Mainz
- Informed (electronic) consent
- Self-reported sufficient proficiency of German language

#### Exclusion criteria

- Self-reported acute, severe psychiatric condition

### Consent provisions {26a, 26b}

Please see the “Ethics and dissemination” section.

## Interventions

### Description of study intervention(s) {11a}

Embedded in an online survey, participants will be exposed to two video vignettes produced specifically for the COPE-Trial. The scripted video vignettes (video A and B) depict a fictional medical consultation between a physician and the parents of a preterm infant. Purpose of the consultation is the disclosure of an unfavorable prognosis on the occasion of a severe intraventricular hemorrhage with parenchymal involvement. Prognostic information in both scenarios is based on data regarding the estimation of outcome from scientific publications [25, 26]. Video A and B differ solely in prognostic framing. Video A exemplifies a more optimistic framing, whereas Video B illustrates a more pessimistic framing. The differing degree of optimism is achieved by varying selected text passages. The number of empathic statements is equal and both vignettes conclude with an identical reassurance of non-abandonment by the physician. Other aspects of the physician-parent communication, e.g., the setting/environment, the consulting physician, the parents, and the content of the consultation, are standardized. The length of both videos is about 3 min each. A female physician was chosen to communicate the prognosis. This reflects the clinical reality of the NICU at the UMC Mainz. The parents are a heterosexual Caucasian couple around 25–30 years old. We have chosen this constellation because their proportion among our local NICU parents and their willingness to participate in research projects is relatively high, without the intention to discriminate against people of other ethnicities or sexual orientation which are also represented on our NICU. The underlying assumption is that the proportion of heterosexual Caucasian parents and couples among the participants would be the largest, and by representing such a parent couple, their identification with the videos and thus the response quality could be optimized.

Prior to exposure to the intervention, participants are provided with a short (approximately 1.5 min) explanatory film contextualizing the medical consultation. A fictional premature infant named Luisa is born with a gestational age of 23 weeks + 5 days due to an amniotic infection syndrome. Luisa experiences an intraventricular hemorrhage on her third day of life. On this occasion, the parents are contacted by telephone by the attending neonatologist. They are asked to come to the unit in person for the purpose of a physician-parent conversation in a timely manner.

### Explanation for the choice of comparators {6b}

Since the optimum framing of prognostic disclosure to parents in the context of complications with an associated unfavorable prognosis in the NICU setting is unknown, we decided to depict two opposing levels of optimism (more optimistic vs. more pessimistic) out of a spectrum of gradations of optimism. As the introduction indicates, these two selected opposing gradations are found in clinical practice as well as in research. Accordingly, this selection is suitable for the purpose of the present project. Stimulus for the choice of comparators as well as for the use of scripted video vignettes as study material originated from a study in adult oncology investigating the influence of more or less optimistic communication on the perception of physician compassion [22]. The selection of the questionnaires chosen to assess physician professionalism and physician compassion was inspired by the aforementioned and one related study [22, 27].

The conception, cinematic realization and application of the video vignettes comply with recommendations by Hillen and van Vliet [28, 29]. Case vignette and scripts were designed with an interdisciplinary team and implemented with professional actors and filmmakers.

### Criteria for discontinuing or modifying allocated interventions {11b}

#### Individual preterm end of study

The individual participant can discontinue the study at any time. The study-related and intervention-related risks for participants are assumed to be minimal. Due to the fact that the study will be conducted as an online survey, among other things, there is no risk of commuting accidents. In addition, the interventions are videos which represent a fictional scenario and do not involve any intervention in the current clinical reality, and the actors as well as the setting (i.e., the filming location) are unknown to the parents. Deliberate care was taken to minimize the relatability to the parents’ own story.

#### Preterm end of entire study

Please see the “Interim analyses {21b}” section.

### Strategies to improve adherence to interventions {11c}

Data collection is performed on 1 day within approximately 30-35 min (time for procession of the online survey) and thus participant adherence can be expected. However, detailed education and transparency regarding the study procedure prior to the online survey are intended to prepare participants as best as possible for study participation and to minimize the risk of non-adherence or drop-out. Education includes the duration of the online survey (baseline questionnaire, single videos, post-intervention questionnaire) and the request to complete the study to the last page in case of participation. The communication of availability for questions or in case of concerns at any time serves the same purpose.

### Relevant concomitant care permitted or prohibited during the trial {11d}

Not applicable, as there is no interference with concomitant care. The video vignettes depict a fictitious scenario and participants’ infants have already been discharged from the NICU.

### Harms {22}, and provisions for ancillary and post-trial care {30}

Watching the videos and the subsequent answering of questions may cause an emotional reaction in parents. However, the risk of experiencing significant distress is considered to be minor. Parents who feel too distressed can discontinue the questionnaire at any time (see the “Individual preterm end of study” paragraph in the “Criteria for discontinuing or modifying allocated interventions {11b}” section). In addition, participants in distress are encouraged to contact the research team to jointly discuss whether their case requires consultative psychological care from a mental health professional in our department.

### Outcomes {12}

#### Primary outcome

1. Parents’ preference with regard to prognostic framing, i.e., the parents’ preference for video A or B after watching both videos.

#### Secondary outcomes and associated hypotheses

1. Parents’ state-anxiety due to/after watching each video: Parental state-anxiety is greater with a more pessimistic prognostic framing than with a more optimistic framing.
2. Parents’ satisfaction with prognostic framing in the first video: Parental satisfaction is greater with a more pessimistic prognostic framing than with a more optimistic framing.
3. Realism of the evaluation of the conveyed prognosis following the first video: With a more pessimistic prognostic framing, the parents’ appraisal of prognostic severity is more realistic than with a more optimistic prognostic framing.
  - Subjective evaluation of the severity of the prognosis: When prognostic framing is more pessimistic, parents indicate a higher degree of severity with regard to the infant’s prognosis (subjective evaluation) when compared to more optimistic framing.
  - Objective ability to recall conveyed prognostic information: When prognostic framing is more pessimistic, parents show less deviation in the recall of conveyed probabilistic data (objective evaluation) with regard to the child’s prognosis when compared to a more optimistic framing.
4. Degree of optimism with regard to the infant’s future (future optimism) following the first video: When prognostic framing is more pessimistic, parents show less optimistic expectations concerning the infant’s future prospects, i.e., its overall survival on the one hand and its survival free from impairment(s) on the other hand, when compared to more optimistic framing.
5. Degree of hope with regard to the infant’s future (future hope) following the first video: When prognostic framing is more pessimistic, parents express less hope with regard to the infant’s future when compared to more optimistic framing.
6. Degree of preparedness by the medical consultation (first video) to make a shared therapeutic decision: When prognostic framing is more pessimistic, parents indicate a higher level of being adequately informed about the prognosis and of feeling prepared for shared therapeutic decision-making when compared to more optimistic framing.
7. General impression of the consulting physician (first video) on parents: A neonatologist conveying a pessimistically framed prognosis makes a better general impression on participants than one conveying it optimistically framed.
8. Professionalism of the consulting physician (first video): Parents assess a neonatologist conveying a pessimistically framed prognosis as more professional than one conveying it optimistically framed.
9. Compassion of the consulting physician (first video): Parents assess a neonatologist conveying a pessimistically framed prognosis as less compassionate than one conveying it optimistically framed.

#### Further outcomes and associated presumptions:

10. Perception of prognostic framing following each video: Parents rate both videos differently in terms of the level of optimism in prognostic framing, whereby prognostic framing in video A is perceived as more optimistic when compared to video B.
11. Parents’ personal preference of gradation of prognostic framing (level of optimism) after watching both videos: Parents’ personal preference of the level of optimism in prognostic framing after watching both videos corresponds to a more nuanced gradation of the direction of prognostic framing indicated via their preference for video A or B.
12. Importance of physician-parent communication in general and of prognostic information in particular (on completion of the survey): Parents rate selected aspects of physician-parent communication in general and of prognostic disclosure in particular as rather important.
13. Burden due to study participation in general and to interventions in particular (on completion of the survey): Parents’ self-assessed burden by participation in the study in general and by watching the videos in particular is low.

A tabular overview of all relevant outcome measures and the time points of assessment thereof (data acquisition) complemented by a detailed description of all collected baseline data, of all outcome measures and of the respective data collection instruments can be found in the “Data collection: outcome measures and assessments” section.

### Participant timeline {13}

#### Study procedures, examination methods, and outcome assessment

Figure 1 provides an overview of the study sequence and corresponds to the participant timeline. Following the recruitment of participants, the online survey based on a two-part questionnaire with embedded scripted video vignettes (interventions) is applied.

#### Sample size rationale {14}

At first, we conservatively assumed that only one parent of each child participates. The data can be arranged in a 2 × 2 table, with columns defining the sequence order “AB” vs. “BA” and rows defining whether the first video was preferred over the second one. Then, the square root of the odds ratio is a period-adjusted estimate of the preference ratio, i.e., a general tendency toward or against preferring the latter video is corrected for. Assuming that there is, in fact, no such period effect, the discriminatory power for detecting a preference ratio of 3:2, based on a two-sided chi-square test with level *a* = 0.05, is 0.80, if 194 individual parent decisions are evaluable (using SAS software’s proc power). Assuming a substantial period effect, such that the first video is preferred with odds 1:1 in sequence group “AB” and with odds 9:4 in group “BA,” the period-adjusted preference ratio results, but then the required case number is 204. Assuming further that 95% of participating parents actually decide and share their preference, 215 families are to be recruited. If we now assume that always both parents participate and that the concordance of the evaluation corresponds to a kappa of 0.5, then at least 153 families would have to be recruited (using the standard formula for cluster randomized trials and multiplying 204 with the variance inflation factor 1.5 results in 306 parents, i.e., in 153 families). A blinded sample size reassessment is planned after 50 to 70 parents. For details, please see the “Interim analyses {21b}” section.

### Participant recruitment {15}

Potential participants, i.e., parents or guardians (individuals or couples), whose preterm infants were born extremely preterm, i.e., with a VLBW (< 1500 g), between 2010 and 2019 and who received postnatal care in our NICU (see “Inclusion criteria” paragraph in the “Eligibility criteria {10}” section) are mainly identified via the electronic database (contact list) of the designated follow-up consultation program intended for former VLWB patients of our NICU, to which the latter are affiliated for the first 2 years after discharge. In addition, the identification of potential participants is complemented by research in the electronic hospital database of the UMC Mainz. Excluding bereaved parents, all parents and guardians are to be invited to participate in our study. Contact with potential participants is established via personal and electronic means. Participants receive a letter with information material (written information) two weeks ahead of a phone call by a research team member (oral information). The material comprises a synopsis of the research project, information concerning data protection regulations, the declaration of consent, and contact details of the research team. The telephone contact is intended to provide a more detailed, in-depth explanation of study-related content and space for queries. The concomitant identification of parents not eligible for participation is a further purpose of the conversation. In addition, potential participants will be contacted personally at neurodevelopmental follow-up appointments, and through a local parents’ mutual support group.

Persons interested in participation contact the research team electronically (e-mail). Parents and guardians are asked to simultaneously include in the electronic feedback to the research team whether one or both parents of the same child will participate. In response, the participants are provided with their personal access data (link and password) for the password-protected online survey. The personal link containing the information which study group the respective participant is assigned to is part of the first e-mail. For data security reasons, the password (a multi-digit access code) is communicated in the second e-mail. Although the recruitment procedure implies contacting and educating parents of the same preterm infant together, during participation they are not treated as an entity. Individual parents are considered equally as related parents of the same child. A special provision in the case of participation of both parents is that they are assigned to the same study group. This is to ensure that the assumed common response tendency (covariance) based on a couple’s shared experience of prematurity is taken into consideration. However, in order to be able to simultaneously investigate sex-specific or parent-specific differences in processing the same lived experience, related parents are marked as such in the data set. This is ensured by the multi-digit access code containing a matching prefix for parents of the same child.

## Assignment of interventions: allocation and blinding

### Sequence generation {16a}, allocation concealment mechanism {16b}, and implementation {16c}

Concealed randomization of participants will be performed in blocks of variable length. Randomization lists will be stratified by the participating parents (single/both) of the same preterm infant, i.e., into the three strata “participation of mother only,” “participation of father only,” and “participation of both parents.” The randomization lists will be generated by JK who is not involved in clinical patient care and data acquisition. With regard to the parent-participants, one list for each stratum will be compiled. Subsequently, sequentially numbered, sealed, and opaque envelopes indicating the respective study group will be prepared by an employee of the UMC Mainz not involved in clinical patient care and data acquisition. Participants will be enrolled by FF. Allocation will be performed by an employee of the UMC Mainz not involved in participant recruitment outside the online survey software and in the order of consent after recruitment of the participants. In case both parents of the same child participate, they receive the same allocation.

### Who will be blinded {17a}, procedure for unblinding if needed {17b}

Actors (video vignettes) were and participants will be blinded to the hypotheses of the study. Research team members (including outcome assessors and data analysts) as well as trial participants cannot be blinded for the intervention sequence. The videos though will not be labelled explicitly as pessimistic or optimistic for the parents and the sequence of interventions will be neutrally labelled. No procedure for unblinding is necessary.

## Data collection: outcome measures and assessments

Table 1 provides an overview of the data acquisition including all important data (enrolment, baseline, outcomes) as well as the respective time points of the assessment thereof. The respective assessments (i.a., all customized questions and validated instruments) are described in detail in the “Data collection: outcome measures and assessments” section under the heading “Assessments: description of instruments used for collection of baseline and outcome data”.

**Table 1:** Overview of time points of exposure to interventions and data acquisition

	Enrolment	Baseline questionnaire	Post-intervention questionnaire				
Time point	*t*_−1_	*t*_0_		*t*_1_		*t*_2_	*t*_end_
**Enrolment**							
Eligibility feedback to RT	●						
Informed consent (electronic)	●						
**Randomization**							
Group allocation	●						
**Baseline data**							
Sociodemographics		●					
Prematurity as special life event		●					
Relevant psychological characteristics		●					
**Interventions**^**a**^			**Video A/B**		**Video B/A**		
**Primary outcome**							
1 Preference video A or B						●	
**Secondary outcomes**							
1 State-anxiety				●		●	
2 Satisfaction (framing)				●			
3 Realism of prognosis evaluation				●			
a Severity of prognosis				●			
b Recall of prognostic information				●			
4 Future optimism				●			
5 Future hope				●			
6 Preparedness for decision-making				●			
7 Physician general impression				●			
8 Physician professionalism				●			
9 Physician compassion				●			
**Further outcomes**							
10 Perception of framing				●		●	
11 Preference gradation of framing						●	
12 Importance of communication							●
13 Burden by study							●

*Notes*. RT: Research team. ^a^The interventions video A and B are embedded in the post-intervention questionnaire. Prior to exposure to the first video (A or B, depending on study group) participants watch a short explanatory film

### Plans for assessment and collection of baseline and outcome data {18a}

Baseline and outcome data will be collected within the framework of the two-part online survey. Entitlement to take part in the online survey (second phase) ensues with the signature of the electronic declaration of consent. Randomization, i.e., balanced random allocation of participants to the two study groups, marks the conclusion of enrolment. Using the individual access data, participants can process the password-protected two-part online survey on the platform SoSci Survey (Dominik Leiner, 2019; URL: https://www.soscisurvey.de). Both parts of the survey are processed directly one after the other, i.e., without any temporal separation. It will take the individual participant an average of 30 to 35 min to complete the two-part online questionnaire. The distinction in two parts is exclusively content-related. The first part of the online survey is identical for both groups. It consists of the assessment of baseline data (baseline questionnaire) followed by the presentation of the identical explanatory film. Subsequently, the two different video vignettes are presented in randomly assigned sequence (cf. Fig. 1, AB vs. BA). The videos are embedded in the post-intervention questionnaire. The latter comprises the identical data acquisition for both groups after the first video (data acquisition *t*_1_), after the second video (data acquisition *t*_2_), and in conclusion (data acquisition *t*_end_).

#### Baseline questionnaire (data acquisition *t*_0_)

Recognizing the interindividual differences that exist between parents may be an important resource in gaining a better understanding of participants’ perceptions and preferences. For this purpose, a selection of relevant participants’ characteristics is assessed as potential factors influencing the parents’ preference for a more optimistic or a more pessimistic prognostic framing as well as the secondary outcomes within the framework of the baseline questionnaire. On the assumption that prematurity can represent a special, even transformative life event for parents [3], the data collection at baseline additionally comprises questions about the parents’ own experience of premature birth as a special life event (adapted from FaBel questionnaire). The additional selection of personality traits assessed, consists of resilience (BRS), (in)tolerance of uncertainty (UGTS), dispositional optimism/pessimism (SOP2), dispositional hope (HHI-D), depressivity and general anxiety (PHQ-4) as well as situational anxiety (STAI-SKD) and anxiety as a personality trait (trait-scale of the STAI). The assessed baseline data serve as the basis for potential subgroup analyses (for details see the “Methods for additional analyses {20b}” section).

#### Post-intervention questionnaire (data acquisition *t*_1_, *t*_2_, *t*_end_)

The post-intervention questionnaire serves the purpose of assessing primary as well as secondary outcome measures. All secondary outcomes are assessed solely after the first video (data acquisition *t*_1_). The primary outcome and the majority of the further outcomes are assessed solely after the second video (data acquisition *t*_2_). One secondary and one further outcome are assessed after each video (data acquisition *t*_1_ and *t*_2_).

##### Primary outcome

1. Parents’ preferences with regard to prognostic framing, i.e., parental preference for video A or B after watching both videos is assessed using the preference for one of the videos A or B as surrogate for different prognostic framing. A dichotomous question (1 = first video; 2 = second video) is used for this purpose. Qualitative complement to the primary outcome (quantitative): In case parents want to substantiate their choice, e.g., if they do not explicitly prefer one of the videos or have a strong preference, a free text field is provided for this purpose.

##### Secondary outcomes

1. Parental state-anxiety is assessed using the German short version (5 items) of the state scale of the State-Trait Anxiety Inventory (STAI-SKD) after each video. The change in state-anxiety due to/after watching the first video is determined as the difference in state-anxiety at time points *t*_0_ and *t*_1_. The change in state-anxiety due to/after watching the second video is assessed analogously. Qualitative complement to the secondary outcome 1 (quantitative): A free text field following the STAI-SKD after each video gives participants the opportunity to describe how they feel after watching the videos in their own choice of words.
2. Parents’ satisfaction with prognostic framing in the first video (customized question) is assessed using a unimodal, fully verbalized 7-point rating scale (1 = not at all satisfied; 7 = very satisfied).
3. Following the first video, the realism of the evaluation of the conveyed prognosis is assessed by the degree of concordance between subjective evaluation and objective ability to reproduce the prognostic information provided: (a) Subjective evaluation of the severity of the prognosis transmitted is assessed using a parent-rated customized statement via a fully verbalized 7-point rating scale (1 = very severe; 7 = not at all severe). (b) The objective ability to reproduce the transmitted information is based on the recall of outcome data in numerical values (relative probabilities in %). Assessment consists in the selection of percentages ranging from 0 to 100 percent in increments of ten (survival) or twenty-five (impairment).
4. The degree of optimism with regard to the infant’s future following the first video is determined by the rating of two customized statements (a) on survival of the complication per se and (b) on survival without impairment via the respective unimodal, fully verbalized 7-point rating scale (1 = not optimistic at all; 7 = very optimistic). It is considered in the analysis that the degree of optimism may depend on the participants’ basic attitude toward life initially assessed using the Scale Optimism-Pessimism-2 (SOP-2).
5. The degree of perceived hope following the first video is assessed rating a customized question on a unimodal, fully verbalized 7-point rating scale (1 = not at all hopeful; 7 = very hopeful). A possible correlation between the individual degree of hope assessed at baseline using the German version of the Herth Hope Index (HHI-D) and the degree of hope following the video is examined.
6. Participants evaluate how well they feel prepared by the medical consultation (first video) to make a shared decision regarding the infant’s treatment (customized question). A fully verbalized 7-point rating scale (1 = not at all prepared; 7 = fully prepared) is used for assessment.
7. Following the first video, the consulting physician’s general impression (customized question: “What is your general impression of the doctor?”) on participants is assessed using a fully verbalized 5-point rating scale (1 = poor; 5 = very good, sum score: range 9–45). The general impression corresponds to a global assessment of the physician on the basis of a widely used evaluation standard (“school grades”) and is supplemented by a more differentiated assessment of individual aspects, i.e., physician characteristics, in two follow-up questions.
8. Following the first video, the consulting physician’s professionalism is assessed by means of the German translation of 7 items of the General Medical Council (GMC) Patient Questionnaire adapted from the original by Campbell et al. [30].
9. Following the first video, the consulting physician’s compassion is evaluated by means of the German translation of the Physician Compassion Questionnaire adapted from the original by Fogarty et al. [31].

##### Further outcomes

10. To capture the perceived differences between both video vignettes in the degree of optimism (customized question), the latter is assessed after each video using a 7-point rating scale (1 = not optimistic at all; 7 = very optimistic). The assessment is the basis for the analysis of intra- as well as inter-group differences concerning the perception of prognostic framing in the videos.
11. The participants’ hypothetic personal preference with regard to framing of an unfavorable prognosis in clinical reality (customized question) is assessed after watching both videos using a unimodal, fully verbalized 7-point rating scale (1 = not at all optimistic; 7 = very optimistic).
12. Concluding the survey, parents are asked to provide an assessment regarding the importance of physician-parent conversations in general and certain contents, i.e., the transmission of prognostic data, in particular. For both topics, 4 customized items are rated using a fully verbalized 5-point rating scale (1 = strongly disagree; 5 = strongly agree).
13. Concluding the survey, parents indicate whether they feel burdened due to study participation in general or due to watching the videos in particular (customized question). The assessment is based on the rating of 2 customized items on a 5-point rating scale (1 = not at all burdened; 7 = very much burdened).

### Assessments: description of instruments used for collection of baseline and outcome data

#### Sociodemographic variables

Sociodemographic variables include age, sex, sociocultural background, highest educational attainment (school, professional), occupation and medical background (general medical and NICU expertise). Additionally, the data acquisition at baseline comprises questions regarding the own family (i.a., family status, number of children) as well as characteristics of the preterm infant (i.a., year of birth, gestational age at birth, birth weight).

#### German version of the Impact on Family Scale (FaBel questionnaire)

The German version of the American “Impact on Family Scale” [32] namely the “Familien-Belastungs-Fragebogen” (FaBel questionnaire) [33] is a self-assessment tool used to assess the impact of a child’s chronic illness or disability on different dimensions of family life. Since early preterm birth can be regarded as a chronic condition on the one hand, and typical complications associated with it often result in impairments on the other, singular items of the FaBel questionnaire were identified as suitable to be selected as a base for the formulation of items on respective topics. In the German version of the scale, parental (dis)agreement with each of the 33 items is assessed using a 4-point Likert scale (1 = strongly agree to 4 = strongly disagree). Each item is assigned to one of five dimensions reflected in the respective number of subscales: everyday and social burden (15 items), personal burden/worries about the future (5 items), financial burden (4 items), burden on siblings (6 items), and problems in coping/mastery (3 items). The average rating of these items corresponds to the score of each subscale. Higher scores in the subscales indicate a higher burden in the respective dimensions, a higher average score of all item scores indicates a higher global familial burden. Testing and evaluation of the German version was performed with a sample of 273 families of children with chronic conditions or disabilities [33]. Factor analysis yielded a five-factor solution instead of a 1-factor plus a 4-factor solution (English version) [33]. The internal consistency for the overall score (*α* = .89) can be rated as good [33]. For the five subscales, reliability can be rated as questionable to good with the internal consistency ranging from *α* = .60 to *α* = .87 [33]. In summary, psychometric testing of the FaBel questionnaire revealed acceptable construct validity (slight deviation from original scale), good internal consistency (Cronbach’s α), very good to excellent scale fit (differential factorial validity), and good discriminant validity [33].

Within the framework of the COPE-Trial, participants will be asked to what extent premature birth has (had) an impact on family life (1 item), family cohesion (1 item), and the family’s financial security (4 items). For each item, parents will indicate on a 4-point Likert scale to what extent they (dis)agree with the respective statement. Five of the six items were inspired by five items of the FaBel questionnaire. The item assessing the impact of prematurity on family cohesion was inspired by the item F12 of the subscale “problems with coping/mastery” of the German version of the FaBel questionnaire. The four items with respect to the impact of prematurity on the family’s instrumental resources were adapted to the items F1-F4 of the subscale “financial burden” of the same scale. Concerning the selection of adapted items in the analysis of results in the present study, each item will be considered individually, with a higher score indicating a higher load regarding the different dimensions covered by the abovementioned items. In addition, an average score can be calculated for the 4 items concerning financial stress. A higher score in this regard also indicates a higher financial burden.

#### German version of the Brief Resilience Scale (BRS)

The Brief Resilience Scale (BRS) is a self-report instrument used to assess an individual’s ability to recuperate from stress despite difficult circumstances [34]. The German version translated, revised, and evaluated by Chmitorz et al. is used in the present study [35, 36]. The short instrument comprises 6 items rated on a 5-point Likert scale (1 = strongly disagree; 5 = strongly agree). The items 1, 3, and 5 are positively phrased whereas the items 2, 4, and 6 are negatively formulated. The latter have to be reversely coded to calculate the mean of all items (range 1–5). Higher average scores (mean) indicate a higher ability to recuperate from stress. Psychometric properties of the German version of the BRS were assessed by use of data from a population-based sample (sample 1) of 1.481 healthy adults aged 18 to 75 from Mainz (Germany) and a representative sample (sample 2) of 1.128 persons from the German general adult population [35, 36]. Differing from previous research (one- and two-factor model), factorial analyses yielded evidence for a method-factor model (both samples). The use of the unidimensional BRS score is however recommended by the authors. Reliability (Cronbach’s *α*) and composite reliability (McDonald’s omega) were rated good in both samples (*α* = .85 and *ω* = .85). Sufficient to fair convergent validity and acceptable to good discriminant validity as well as construct validity were demonstrated [35, 36]. Normative data is available for a German general population sample [36].

#### German Uncertainty Tolerance Scale (Ungewissheitstoleranzskala, UGTS)

The Ungewissheitstoleranzskala (UGTS), i.e., the German Uncertainty Tolerance Scale, enables the differentiation of persons tolerant and persons intolerant to uncertainty [37]. The unidimensional self-rating scale consists of 8 items phrased in the first person used to assess the respondent’s evaluation of and/or coping with uncertain situations (e.g., “I like unexpected surprise”). Each item is rated on a 6-point rating scale (6 = strongly agree; 1 = strongly disagree). Five items describe uncertainty tolerance whereas 3 items (items 2, 5, and 8) describe uncertainty intolerance. The latter have to be recoded in order to calculate the mean (range 1–6). Higher scores indicate higher uncertainty tolerance, whereas lower scores indicate higher uncertainty intolerance. The internal consistency ranges from Cronbach’s *α* = .66 to .72 and can be rated as acceptable to good [37]. The UGTS was based on a one-factor model which could be confirmed in multiple studies, and data concerning construct validity and differential validity are available [37]. For the UGTS, no normative data, but mean values and standard deviations for certain samples are available [37].

#### German Scale Optimism-Pessimism-2 (SOP2)

The SOP2 is a short self-rating instrument consisting of two items [38, 39]. The two-dimensional scale is used to assess the construct optimism-pessimism, i.e., a respondents’ dispositional optimism and/or pessimism. On a 7-point Likert scale, respondents indicate how optimistic (1 = not at all optimistic; 7 = very optimistic) or pessimistic (1 = not at all pessimistic; 7 = very pessimistic) they are in general. Precondition to calculate the average score SOP2 (mean) is the recoding of the item pessimism (range 1–7). Lower SOP2 scores indicate higher pessimism and higher scores signify higher optimism. There is no consensus in the literature concerning the dimensionality (one- vs. two-dimensional) of the conceptualization of the construct optimism-pessimism. Consistent with data from sample 1, the SOP2 was based on a general factor model [38, 39]. The SOP2 was developed and validated within three studies (series) using three samples - one quota sample with two waves (sample 1; *N*_first wave_ = 539, *N*_second wave_ = 338), one quota sample from the internet (sample 2; *N* = 741), and one large sample representative for the German adult population (sample 3; *N* = 1134)—by Kemper et al. [38, 39] The composite reliability (McDonald’s ω) of the SOP2 was rated sufficient to good (*ω* = .74–.83) [38, 39]. Data concerning convergent and divergent validity (e.g., strong correlation with LOT-R and life satisfaction), and expected correlations confirming the construct validity are available [38, 39]. In large-scale studies, psychometric evidence for the German version of the SOP2 was shown [40]. Normative data exist for different groups separated by age, gender, and education for a population-representative sample (resident population in Germany > 18 years) [38, 39].

#### German version of the Herth Hope Index (HHI-D)

The Herth Hope Index is a 12-item self-report instrument to assess the construct of hope [41]. Various dimensions of hope as well as the coexistence of generalized as well as particularized hopes are reflected in the 12 statements (items). The items are rated on a 4-point Likert scale (1 = strongly disagree, 4 = strongly agree). The individual level of hope is assessed by calculating the total score (range 12–48). In doing so, the negatively phrased items 3 and 6 have to be recoded. Higher scores indicate a higher level of hope, lower scores a lower level of hope. The original version of the HHI was translated by Geiser et al. and validated with a sample of cancer patients in treatment at the University Hospital of Bonn, Germany (*n* = 192) [42]. The original 3-factor structure of the HHI could not be replicated in the sample and a 1-factor structure was proposed [42]. That being the case, it is recommended that only the total score be used to indicate an individual’s level of hope. The internal consistency for the overall score based on the German sample is *α* = .82 and can be rated as good [42]. Test-retest reliability can equally be rated as good (*α* = .80) [42]. Data concerning solid convergent validity (e.g., significant correlation of the HHI-D sum score with LOT subscale optimism and the subscale of pessimism) and expected correlations confirming the construct validity are available [42].

#### German version of the 4-item Patient Health Questionnaire-4 (PHQ-4)

The PHQ-4 is a 4-item self-report questionnaire consisting of two subscales, namely the 2-item depression scale PHQ-2 and the 2-item anxiety scale GAD-2 [43]. The ultra-brief instrument is used to assess depressivity (depressive disorders) and anxiety. By rating the 4 items of the questionnaire (e.g., “feeling down, depresses or hopeless”) on a 4-point rating scale (0 = not at all, 1 = several days, 2 = more than half the days, 4 = nearly every day), respondents indicate the frequency of their occurrence within the past 2 weeks. The German version of the PHQ-4 has been validated and standardized in the general population by Löwe et al. [44] with a sample of 5030 participants. Confirmatory factor analyses showed a very good fit for a two-factor model. The internal consistency of the subscale PHQ-2 (*α* = .78) and GAD-2 (*α* = .75) as well as of the average score (*α* = .82) can be rated as acceptable considering the shortness of the subscales as well as of the questionnaire. Study findings suggest good construct validity (correlation with selection of self-report scales and with demographic risk factors for anxiety and depression) in the general population. On the basis of available studies, convergent, divergent, and factorial validity of the PHQ-4 can be rated as good [43, 44]. Normative data is available for a German general population reference group [44].

#### German version of the trait scale (STAI-T) of the State-Trait Anxiety Inventory (STAI)

The State-Trait Anxiety Inventory is a self-rating instrument consisting of two separate questionnaires with 20 items each [45]. One of the questionnaires, namely the state-scale of the STAI, serves the purpose of determining anxiety as a state, i.e., the respondent’s current, situational anxiety. The second questionnaire, the trait-scale, is used to assess anxiety as a trait, i.e., the respondent’s dispositional anxiety (independent of a particular situation). In both questionnaires, the rating of 20 brief self-statements on a 4-point rating scale is required. In the case of the present study, only the trait-scale is applied. To assess situational anxiety, a shorter version of the original state-scale is used (see next section). Verbal anchoring differs in both questionnaires and describes frequency dimensions in the case of the traitscale (1 = almost never, 2 = sometimes, 3 = often, 4 = almost always). Thirteen of the items describe characteristics positively formulated, i.e., describing presence of anxiety. The remaining 7 are negatively phrased, i.e., indicating absence of anxiety. The latter have to be recoded before calculating the sum of all 20 scores on the individual items. The total score (range 20–80) corresponds to a global assessment of the respondent’s dispositional anxiety. Higher scores indicate a higher trait-anxiety. The original version of the STAI-T was translated to German by Laux et al. and validated with data from multiple subsamples (healthy persons as well as collectives of people with a mental health condition) [46]. Internal consistency of the average STAI-T score is *α* = .90 and can be rated as good to excellent [46]. The construct validity of the STAI is confirmed by high correlations with scales related to the construct [46]. Normative data are available for different groups (students, different clinical groups) separated by age and gender [46].

#### German (5-item) short version of the state scale of the State-Trait Anxiety Inventory (STAI-SKD)

The short version of the state-scale of the STAI is used to assess the respondent’s situational anxiety [47]. The STAI-SKD consists of 5 items concerning the respondent’s current state of tension, excitement, nervousness, fear, and worry rated on a 4-point rating scale (1 = not at all; 4 = very much so). Compared to the original state-scale comprising 20 items, all 5 items of the short version are worded toward the presence of anxiety. The extraction of the short version from the original STAI state-scale was based on a first sample of university students (*N* = 65). Confirmatory factor analysis yielded a two-factor structure in a second sample of university students (*N* = 191). In a third sample of university students (*N* = 80), the construct validity of the STAI-SKD (additional to discriminant validity assessed in second sample) and its sensitivity to change were assessed [47]. The expected two-factor structure of the STAI-SKD with the two factors emotionality and worry was confirmed [47]. However, due to the high correlation of both factors, the use of the total score is recommended to indicate a respondent’s state-anxiety. The internal consistency for the overall score based on three German samples of university students ranges from *α* = .76 to *α* = .85 and can be rated acceptable to good [47]. The instrument’s sensitivity to change was confirmed [47]. That is determinant for its use in the framework of the present study. Expected correlations with other parameters confirming construct validity are available [47].

#### Physician Professionalism—GMC Patient Questionnaire

The Physician Professionalism Questionnaire corresponds to a selection of a 7-item questionnaire adapted from the General Medical Council (GMC) Patient Questionnaire [30]. The original patient questionnaire consists of 18 items, 11 of which are used to measure a patient’s perception of the physician’s performance. To evaluate the physician professionalism within the framework of the present study, from these 11 items the 7 respective items (3a–g) were selected. The selection of items was adapted from Tanco et al. [22, 27]. Professionalism will be assessed via the respondent’s evaluation of the attending physician’s politeness, of the pleasantness of the atmosphere in his/her presence, of the physician’s ability to listen to the patient, to assess the patient’s condition, to explain the condition and its treatment to the patient, and to involve the patient in the treatment decision. Analog to the original questionnaire, the evaluation will be realized by the use of a verbally and numerically anchored 5-point rating scale with the scale points poor (= 1), less than satisfactory (=2), satisfactory (=3), good (=4), very good (= 5). In the context of the present study, higher scores on individual items will be interpreted as greater competence in the trait in question. In addition, a total score (range 7–35) of all items can be calculated with a higher sum score corresponding to higher global professionalism. Analog to the reference studies [22, 27], the cut-off for professionalism will be set at ≥ 4. The internal consistency of the original 9-item questionnaire can be rated as excellent (*α* = .962) [30]. Translation by a native speaker as well as retranslation have occurred. The phrasing of the items was adapted to the context of the study (e.g., use of parents instead of patients).

#### Physician Compassion Questionnaire

The applied version of the Physician Compassion Questionnaire comprises 5 items used to assess physician compassion. The 5 items correspond to five dimensions of compassion evaluated on a 10-point numerical rating scale each (polarity profiles with 10 scale points each). The five dimensions reflected in the questionnaire are as follows: warm-cold, pleasant-unpleasant, compassionate-distant, sensitive-insensitive, caring-uncaring. To determine the global compassion of the consulting physician, the cumulative value (sum score) of the 5 scales is calculated (range 5–50). Higher scores indicate higher physician compassion. For the purpose of the present study, the version of the questionnaire used by Tanco et al. [22, 27] was translated to German and retranslated by a native speaker. Concerning the original instrument developed by Fogarty et al. [31], internal consistency can be rated as excellent (*α* = .92).

### Plans to promote participant retention and complete follow-up {18b}

The provision of thorough information (transparency) about the study, the availability of the research team for queries at any time, and the possibility of flexible processing of the online survey in terms of time and space are intended to promote participant retention and completion of the questionnaire.

### Data management and quality assurance {19}

Data management and quality assurance will follow the FAIR principles [48]. The management of data from project planning to storage and accessibility (flow of the study data) will be documented and updated by the principal investigator (FF) on an ongoing basis. FF will further be responsible for the assessment and documentation of missing data. In the case of missing data, parents will be re-contacted and missing values will be obtained.

The following data with regard to the course of the study will additionally be assessed: Number of parents eligible and approached, number of parents excluded (including reasons for exclusion: participation refused/rejected, exclusion criteria met, other reasons), number of parents included and number of parents randomized to each study group/respective scenario, number of parents discontinuing the trial, number of parents included in final analysis (including reasons for non-analysis).

## Statistical methods

### Statistical methods for primary and secondary outcomes {20a}

SPSS in the latest version (IBM, Armonk, NY, USA) and the R software (R Core Team, Vienna, Austria; URL https://www.R-project.org/) will be used to conduct all statistical analyses.

#### Descriptives

For all variables (baseline characteristics, primary, secondary, and further outcomes), standard descriptive statistics including means, medians, standard deviations, ranges, and proportions will be determined. Baseline characteristics will be reported for both study groups. In addition, for the primary, the secondary as well as for the further outcomes, appropriate effect estimates will be reported with 95% confidence intervals.

#### Inferential statistics

The primary outcome is the answer to the binary question whether a participant prefers the first or second of the two videos (presented in different sequence in both study groups). We will present results as 2 by 2 table of preference (first or second video) vs the sequence of presentation (AB and BA). Then the square root of the odds ratio will be used as an effect measure which is adjusted for systematic presentation order effect (either for the first or for the latter video). In order to also adjust for dependent response within families this odds ratio and a 95% confidence interval will be calculated by fitting a marginal logistic model for dependent response. For this purpose, parents are considered as cluster of size 1 or 2. The exponentiated halved coefficient of the factor “sequence” (A then B coded as 0, B then A coded as 1) is used as the period-corrected estimate of the odds for preference for intervention A. The test of the null hypothesis is performed as Wald chi-square test at the two-sided 0.05 level.

Secondary outcomes will be analyzed by fitting appropriate linear mixed models. In particular, for outcome scales assessing each parent’s attitude at one instance only, one random intercept term at a family level will be entered. For the further outcome that is observed after each video, the change between both observations *t*_2_ vs. *t*_1_ is modelled and the half-difference between sequence groups is taken as period-adjusted effect measure and is reported with 95% confidence intervals.

#### Effect size

The effect size is the odds for preference for intervention A. For sample size rationale, we considered an effect size of 3:2 as a plausible relevant effect size.

For calculation of standardized effect sizes for quantitative secondary outcomes, the outcomes are rescaled such that the random intercept variance at a parent level and the residual variance add up to one, and will be referred to as Cohen’s *d*.

### Interim analyses {21b}

No interim analysis for treatment effect is planned. Because the period effect (i.e., the overall proportion preferring the first video), the proportion of families participating with both parents, the concordance between parents, and the proportion of evaluable answers are unknown, a blinded (using data on preference of video 1, parents’ ID, sex, and neutrally labelled sequence group) sample size reassessment is planned after recruitment of 50 to 70 patients.

### Methods for additional analyses (e.g., subgroup analyses) {20b}

Details will be fixed in a statistical analysis plan after a blind data review. Potentially prognostic factors for the preferential attitude of parents will be analyzed by entering them as additional explanatory variables into the marginal logistic regression model described for primary analysis. Similarly, the mixed linear regression models envisaged for secondary outcomes will be extended by entering potential predictors. Selected variables will be investigated each at a time (univariable analysis) and in a multivariable model. Further variables will be analyzed exploratorily in univariable and multivariable models in a forward step-up procedure.

### Methods in analysis to handle protocol non-adherence and any statistical methods to handle missing data {20c}

Concerning the primary endpoint, participants are asked to decide their preference for one of the two videos after having viewed both. Participants have the possibility to complement their choice by adding an explanation in case of indecision or for clarification in a free text field following the assessment of the video preference, but no option to respond “no preference.” We expect only few participants to refuse answering by discontinuing the online questionnaire. All observations with missing primary outcome will be discarded independent of the reason, because they contribute practically no information. Possible consequences toward loss of power will be addressed through the blinded sample size reassessment.

For secondary outcomes, missing values on item level will be handled according to the guidance given by the authors of the respective scale. Missing values on a scale level either on outcomes or in explanatory variables will be handled by complete case analysis, if this leads to less than 5% case exclusion or by multiple imputation using R package mice [49].

### Plans to give access to the full protocol, participant-level data, and statistical code {31c}

Please see the “Ethics and dissemination” section.

## Oversight and monitoring

### Composition of the coordinating center and trial steering committee {5d}, data monitoring committee (DMC) {21a}, and plans for auditing trial conduct {23}

Quality assurance is ensured by the interdisciplinary research team. The COPE-Trial and its respective interventions (video vignettes) are non-medical and expected to be of minimal risk for participants. Consequently, no safety concerns, overwhelming benefit, or futility are expected and a DMC will not be implemented. Therefore, no external auditing is planned.

## Discussion

### Implications

The COPE-Trial addresses the question which framing of prognostic disclosure is preferred by parents of preterm infants in the context of a severe complication. More detailed knowledge of parents’ preferences is of relevance, as it may contribute to improving the quality of care in neonatology in the sense of parent-centered, personalized communication in the long term. Of scientific interest is whether and how communication can be tailored to parents’ needs and preferences in the future. Parents of preterm infants can provide valuable support thanks to their own lived experience of preterm birth and the associated challenges. We expect that a better understanding of parents’ preferences concerning prognostic disclosure will be of great value in tailoring conversations to parental needs. This may set the stage for the best possible parental coping.

### Limitations

A first possible limitation might be a selection bias due to the (multi-step) recruitment approach, to the eligibility criteria (esp. language skills) or to the intrinsic motivation to participate (esp. gender differences). Consequently, the heterogeneity of the study population might not be reflected in the study collective which can in turn limit the generalizability of the findings. In addition, even though the recruitment procedure chosen for this study allows for a heterogeneous sample, i.e., the mapping of different perspectives, by including families with children with very different outcomes, e.g., with or without physical or cognitive impairments of different degrees, at the same time, certain perspectives are underrepresented. For example, despite the knowledge of the value of the perspective of bereaved parents, it was decided to approach the latter in a separate future study.

Additionally, the personal data and psychological characteristics of parents assessed as part of the baseline (baseline questionnaire) can only be considered a selective sample of potential factors that influence parents’ preference and perception, and can consequently be considered predictors thereof. Further potentially influencing factors, such as the parental worldview and the impact of the own infant’s biography or the own NICU experience, respectively, cannot be comprehensively illuminated in the framework of this trial due to the scope and design of the present study (quantitative vs. qualitative).

The video vignettes do not reflect the full range of prognostic framing, that is the entire spectrum/all gradations of levels of optimism or possible manners of disclosure of unfavorable prognoses in clinical practice, but only two opposite extracts thereof. In addition, the framing of communication in the two videos intentionally differs mainly at the level of verbal communication. However, it must be assumed that participants might also perceive a difference at the level of nonverbal communication (voice, facial expressions). This is due to the natural convergence of content and manner of communication (coherence) occurring when a complex and uncertain message is communicated in an empathetic way. In addition, the sex of the physician is not randomized in the two videos. Depending on life experience and family role models of the parents, the gender of the physician could though potentially influence the parental perception of and preference for one video more than the prognostic framing itself. Moreover, neither the physician nor the parents shown in the videos have a migration background, i.e., they do not reflect the diversity of ethnic backgrounds met in (clinical) reality. This could lead to some participants not being able to identify with the scenario or to not consider the videos as reflection of their (clinical) reality. As a consequence, this could also influence the results.

Finally, the generalizability of our findings is limited by having confined to present only two video vignettes overall as well as by the single-center setting. Note, however, that the choice of vignettes was specifically based on findings from the literature and on the experience of medical professionals (neonatologists) of the NICU at the UMC Mainz. For the present project, we decided on surveying solely parents and guardians of former patients of our center for the following reasons: On the one hand, we assume that knowledge of the contextual factors (e.g., communication practice of practicing physicians, health care practice, parental collective) at our research site will help us to better understand and contextualize our research findings. On the other hand, we regard our study as a starting point for exploring feasibility of various design features before extending it to other research sites.

### Strengths

One of the strengths of the present project consists of the use of validated psychological instruments and consideration of test-theoretical findings in the development of study-specific questions. The project is also characterized by the high degree of standardization of the videos. Furthermore, the comparability of both study groups is guaranteed by use of the identical baseline questionnaire, explanatory film, and post-intervention questionnaire.

A further strength of the project is that it may pave the way toward greater involvement of parents in neonatal care research [50–52]. The COPE-Trial aims to examine, on the one hand, the parents’ preferences concerning the framing of disclosure of an unfavorable prognosis (optimistic vs. pessimistic), and on the other hand, to explore potential factors influencing the parents’ preference as well as parents’ perception of, e.g., the prognosis itself. In order to ensure that the perspectives of both communication partners of physician-parent conversations in the context of the NICU are investigated in a comparable way and with the same thematic focus on prognostic framing (preferences and effects), it is planned to conduct an exploratory trial complementary to the present project to investigate the neonatal doctors’ perspective analogous to and alongside the ones of the NICU parents’ (note: The study protocol of the complementary “COPE-Trial with neonatal doctors” will also be made available upon its completion). The almost analogous conduct of the study with parents with the lived experience of prematurity as well as with the clinical actors allows a direct comparison of both perspectives. The study may therefore contribute to determine the coherence of physician’s assessment of parental needs and preferences and the reality of parental need and preferences. Study findings may contribute to increasing consideration of individual parental needs and preferences with regard to physician-parent communication in neonatology and may help improve the quality of care in neonatology by strengthening a parent-centered approach in this particularly challenging medical field.

## Ethics and dissemination

### Research ethics approval {24} and protocol amendments {25}

The study protocol including the data management as well as one amendment to the study protocol were approved by the Ethics Committee of the Medical Association of Rhineland-Palatinate (Ethik-Kommission der Landesaerztekammer Rheinland-Pfalz), Mainz, Germany (reference number of the COPE-Trial: 2019-14586). The COPE-Trial (original protocol and one substantial amendment) was registered at the German Clinical Trials Register (www.drks.de/DRKS00024466) prior to the commencement of participant recruitment. No further amendments are planned. Any additions and changes to the approved protocol would have to be submitted to the aforementioned Ethics Committee for review. Amendments would require a specification of reasons and can be considered part of the protocol upon signature by an authorized person. Substantial changes would require a new positive vote from the respective Ethics Committee. Important changes to the protocol will be communicated to the responsible Ethics Committee via a designated online platform and will further be added online to the German Clinical Trials Register (www.drks.de/DRKS00024466). In case of important modifications, we will also add the final version of the study protocol as an appendix when publishing the results.

### Consent or assent {26a}, and confidentiality {27}

The trial is conducted in accordance with national and international ethical and legal standards currently applicable (e.g., the Declaration of Helsinki in its latest German version). The legal basis for the protection of personal data is the European General Data Protection Regulation (GDPR). The processing of personal data is performed only after effective declaration of consent (Art. 6 para. 1 letter c) DSGVO).

All parents or legal guardians of infants born preterm (eligible participants) will be informed personally. Written and oral information regarding background and objectives of the study, study procedure, and participation (i.a., eligibility criteria) as well as data protection regulations and (electronical) declaration of consent will be provided to both parents or legal guardians prior to participation. Participants are encouraged to communicate additional questions to the research team during the personal phone call or at any other point in time. Participation is granted upon consent. Electronic, informed consent to participate will be obtained from all participants. In cases that consent is not obtained, the participants are not proficient in German, or the participant suffers from an acute psychiatric illness, participation will be declined. Participants may withdraw from the study at any time without further explanation and without negative consequences. Participants have the right to obtain information on their personal data at any time (including the provision of a copy free of charge) and to request restriction, transfer, correction, or deletion of their data. Furthermore, they may object to the processing of their data at any time (Art. 13-21 DSGVO). SoSci Survey (Dominik Leiner, 2019; https://www.soscisurvey.de), a professional tool for online questionnaires developed in Munich, Germany, will be used for data collection. SoSci Survey allows secure surveys in accordance with data protection regulations. The tool is GDPR compliant and data transmission is fully SSL-encrypted. Collection, processing, analysis, and storage of study-related data will be conducted in pseudonymized form. All data collected are archived on data storage devices, processed exclusively on password-protected computers, and are subsequently stored safely and securely for 10 years at the Center for Paediatri and Adolescent Medicine at the UMC Mainz. Personal data and study data will be stored separately. All personal data and study data, that is answers provided by the participants in the course of the study, will be treated with absolute confidentiality. Study data is in the first instance collected digitally by means of the online survey (questionnaire). Subsequently, a code is assigned to each patient. The study data is stored digitally in conjunction with the patient code. The pseudonymization code is stored in the custody of an independent employee of the UMC Mainz, who is not directly involved in the study. The publication of study results occurs exclusively in anonymous form.

### Additional consent provisions for and collection, evaluation, and storage of participant data and biological specimens {26b; 33}

Not applicable, no collection of data of this scope (e.g., biological specimens) is planned.

### Access to data {29}

Only the principal investigator and members of the research team directly involved in the study can access the data collected (final trial dataset) and are responsible for the evaluation thereof. For data protection reasons, participant-level data will not be accessible to third parties.

### Dissemination policy {31a}

The final study report will be compiled by the principal investigators and forwarded to the ethics committee. Results will be published through peer-reviewed publications. The study protocol and the study findings will as well be disseminated through academic journal articles as well as through presentations at appropriate scientific congresses, and possibly through advanced trainings for neonatal doctors and medical students. Eligible data (scripts for video vignettes and online questionnaire) will be made accessible via adequate repositories (as results databases). Study findings will also be communicated in a simplified format to interested participants.

The COPE-Trial (conception, implementation, analysis, and compilation of findings) is part of a cumulative medical doctoral thesis and is supported by the DFG-Research Training Group 2015/2 “Life Sciences **–** Life Writing,” UMC Mainz, within the framework of a PhD-fellowship.

### Plans to give access to the full protocol, participant-level data, and statistical code {31c}

Data sharing is not applicable to this article as no datasets were yet generated or analyzed during the current study. Access to the full study protocol (detailed long version; in German or English), to supplemental material (in German or English) such as the scripts for cinematic implementation, the respective videos (explanatory film and two video vignettes), the online questionnaire (PDF or XML/JSON/CSV; in German or English) and the final statistical analysis plan/code will be provided upon reasonable request. For data protection reasons, participant-level data will not be disclosed to third parties.

## Trial status

The recruitment of participants was commenced on June 1, 2021, i.e., after the manuscript’s first submission for publication. Following the predefined recruitment procedure, participant recruitment is planned to be completed within approximately 4 months. The protocol version number is 3.0. The date of the protocol version is February 05th, 2021.

## Supplementary Information

**Additional file 1.** COPE-Trial_SPIRIT-Checklist_V02.docx; Version 2 of the completed SPIRIT-Checklist for the revised manuscript of the COPE-Trial

## Abbreviations

DMC: Data Monitoring Committee
DRKS: Deutsches Register Klinischer Studien
GMC: General Medical Council
IMBEI: Institute of Medical Biostatistics, Epidemiology and Informatics
NICU: Neonatal Intensive Care Unit
RCT: Randomized Controlled Trial
RT: Research Team
UMC Mainz: University Medical Center of the Johannes Gutenberg-University Mainz
VLBW: Very Low Birth Weight
